# Microarray Analysis Reveals the Molecular Basis of Antiarthritic Activity of Huo-Luo-Xiao-Ling Dan

**DOI:** 10.1155/2013/524746

**Published:** 2013-07-30

**Authors:** Hua Yu, David Y.-W. Lee, Siddaraju M. Nanjundaiah, Shivaprasad H. Venkatesha, Brian M. Berman, Kamal D. Moudgil

**Affiliations:** ^1^Department of Microbiology and Immunology, University of Maryland School of Medicine, HSF-1, Suite 380, 685 West Baltimore Street, Baltimore, MD 21201, USA; ^2^Mailman Research Center, McLean Hospital, Harvard Medical School, Belmont, MA 02478, USA; ^3^Center for Integrative Medicine, University of Maryland School of Medicine, East Hall, 520 W. Lombard Street, Baltimore, MD 21201, USA; ^4^Division of Rheumatology, Department of Medicine, University of Maryland School of Medicine, Baltimore, MD 21201, USA

## Abstract

Rheumatoid arthritis (RA) is a chronic inflammatory disease of autoimmune origin. Huo-luo-xiao-ling dan (HLXL) is an herbal mixture that has been used in traditional Chinese medicine over several decades to treat chronic inflammatory diseases including RA. However, the mechanism of the anti-arthritic action of this herbal remedy is poorly understood at the molecular level. In this study, we determined by microarray analysis the effects of HLXL on the global gene expression profile of the draining lymph node cells (LNC) in the rat adjuvant arthritis (AA) model of human RA. In LNC restimulated in vitro with the disease-related antigen mycobacterial heat-shock protein 65 (Bhsp65), 84 differentially expressed genes (DEG) (64 upregulated and 20 downregulated) versus 120 DEG (94 upregulated and 26 downregulated) were identified in HLXL-treated versus vehicle (Water)-treated rats, respectively, and 62 DEG (45 upregulated and 17 downregulated) were shared between the two groups. The most affected pathways in response to HLXL treatment included immune response, inflammation, cellular proliferation and apoptosis, and metabolic processes, many of which are directly relevant to arthritis pathogenesis. These results would advance our understanding of the mechanisms underlying the anti-arthritic activity of HLXL.

## 1. Introduction

Huo-luo-xiao-ling dan (HLXL) is an herbal formula based on the principles of traditional Chinese medicine (TCM) [1, 2]. HLXL has long been in use in China for the treatment of a variety of inflammation-mediated disorders, including rheumatoid arthritis (RA). We have previously reported that HLXL can suppress ongoing experimental arthritis in the rat, and this protective effect involved modulation of antigen-induced immunological and biochemical mediators of inflammation [3–6]. Multiple cellular and molecular events involving different functional pathways drive the pathogenesis of arthritis [7, 8]. In this context, we reasoned that examination of transcriptional events in the draining lymph node cells (LNC) using microarrays and large-scale gene expression analysis might be of help in determining concurrent changes in different effector pathways during disease as well as following treatment. In fact, microarray analysis has previously been employed to analyze the correlation of cold and heat patterns with changes in gene expression in patients with RA [9, 10] and to examine the effects of treatment of RA with conventional antiarthritic drugs [11–13].

To gain an insight into the molecular events mediating the beneficial effects of HLXL in adjuvant arthritis (AA), we performed microarray gene expression profiling of the LNC of rats treated with HLXL (experimental group) or Water (control group). The choice of LNC is based on the significance of the draining lymph nodes in the pathogenesis of AA in the Lewis rats. The induction of AA after Mtb injection involves the priming of potentially pathogenic T cells within the draining lymph nodes, followed by the migration of these primed T cells into the joints, leading to the development of arthritis [14–17]. Furthermore, the pathogenesis of arthritis involves not only lymphoid cells but also myeloid-lineage cells [7, 8]. Therefore, we tested bulk LNC instead of purified T cells.

## 2. Materials and Methods

### 2.1. Animals

Male Lewis (LEW/Hsd) (RT.1^l^) rats (5 to 6 weeks old) were obtained from Harlan Sprague-Dawley (HSD) (Indianapolis, IN, USA) and then housed in the animal care facility of the University of Maryland School of Medicine, Baltimore, MD, USA. All experimental procedures performed on these rats were in accordance with the guidelines of the Institutional Animal Care and Use Committee (IACUC).

### 2.2. Huo-Luo-Xiao-Ling Dan (HLXL)

HLXL used in this study is a defined mixture of 11 herbs, and the constituents of HLXL preparations used in those studies and the present study were thoroughly characterized by HPLC fingerprinting as described in our previous studies [1–6]. The toxicity assessment of this herbal product has been described elsewhere [1–3]. Our earlier studies indicated that HLXL (2.3 g/kg, oral feeding) has significant anti-inflammatory and antiarthritic effects in the rat adjuvant arthritis (AA) model of human RA [3–6].

### 2.3. Induction and Grading of Adjuvant Arthritis (AA) and HLXL Treatment of Arthritic Rats

LEW rats were immunized subcutaneously (s.c.) with 2 mg/rat heat-killed *M. tuberculosis* H37Ra (Mtb) (Difco, Detroit, MI, USA) in 200 *μ*L of mineral oil (Sigma-Aldrich) at the base of the tail. At the onset of arthritis, these rats were randomly divided into two groups (experimental and control). HLXL was suspended in Water and fed (2.3 g/kg) to rats using a gavage needle (FNC-16-3, Kant Scientific Corporation, Torrington, CT, USA). The experimental group of rats (T_H_) was daily fed HLXL beginning within 24 h of disease onset (d 10-11) and then continued for 7 days. The control group of rats (T_W_) received Water (the vehicle) by the same procedure. Untreated arthritic rats (T_0_) served as controls for both T_H_ and T_W_. All rats were examined and graded regularly for the severity of arthritis as described earlier [18].

### 2.4. LNC Culture and RNA Extraction

The draining lymph nodes (LN) were harvested from HLXL-treated and vehicle (Water-) treated arthritic LEW rats after 7 days of treatment as described previously [4–6]. A single cell suspension of LNC was cultured at 37°C for 24 h in a six-well plate (5 × 10^6^ cells/well) in serum-free HL-1 medium (Lonza, Walkersville, MD, USA) with or without Bhsp65 (5 *μ*g/mL) [5, 19, 20]. Thereafter, total RNA was extracted from cells using Trizol reagent (Invitrogen, Carlsbad, CA, USA) and further purified using an affinity resin column (RNeasy Mini Kit, Qiagen Ltd, Crawley, UK). The concentration and quality of RNA were measured using NanoDrop ND-1000 (NanoDrop Technologies, Wilmington, DE, USA). The integrity and purity of the total RNA and cRNA were analyzed using a Bioanalyzer 2100 and RNA kit 6000 LabChip (Agilent Technologies). The 28S/18S ratio of total RNA ranged from 2.0 to 2.4, and RNA integrity number (RIN) ranged from 8.3 to 9.5. This RNA preparation was then used for further generation of cRNA for testing in microarrays.

### 2.5. cRNA Synthesis and Probe Array Hybridization

The biotin-labeled cRNA was synthesized according to the 2-step amplification protocol outlined by the manufacturer (Illumina) with 100 ng of total RNA as input material. Then, the biotin-labeled product was hybridized onto an Illumina RatRef-12 Expression BeadChip (a genome-wide array with 22,000 probes) for whole genome expression profiling according to Illumina's Direct Hybridization Assay protocol. The assays were performed in the Biopolymer-Genomics Core of UMB. Three independent experiments, that is, 3 gene chips/group of biological replicates, were performed. Thereafter, the signal was scanned on iScan.

### 2.6. Microarray Data Analysis

The signal intensity was calculated with the Illumina BeadStudio software. Expression data were processed with GeneSpring (Agilent Technologies, Santa Clara, CA, USA) for normalization and pair-wise analysis. Briefly, data files (with the extension CEL) were uploaded in the GeneSpring GX program and grouped. Then the data was processed using the “Default Guided Workflow” to calculate Robust Multiarray Average (RMA) and Baseline Transformation to Median of all Samples. Also performed was quality control (QC) for background check. Following that the lowest 20 percentile of all the intensity values on the raw signal values was filtered, and the analysis for significance was performed by setting the cutoff limits based on false discovery rate (FDR) and “fold change”. For the gene expression profile in vitro in response to Bhsp65, the expression level of genes induced by Bhsp65 restimulation of LNC was compared with that of the baseline (LNC cultured in medium only). For analysis of ex vivo gene expression profile of arthritic rats, the gene expression of LNC in medium of HLXL-treated (T_H_) and Water-treated (T_W_) rats was compared. Furthermore, the expression profile of untreated arthritic rats (T_0_ group) was compared separately with T_H_/T_W_ group. The significance (*P* value) of differentially expressed genes (DEG) was determined by unpaired *t*-test. Fold change in gene expression >2.0 and *P* value < 0.05 corrected with Benjamin-Hochberg were used to estimate the FDR as the threshold for identifying DEG. For comparing the expression of DEG shared between T_H_ and T_W_, either ex vivo or in vitro, significantly modulated genes were selected by setting ∆ fold change to >0.5.

### 2.7. Quantitative Real-Time PCR (qPCR)

A total of 1 *μ*g of RNA used for the microarray analysis was reverse-transcribed to cDNA using iScript cDNA synthesis kit (Bio-Rad), and the resulting cDNA (1 : 10 dilution) was amplified and assayed by qPCR. For qPCR, SYBR Green (PCR Master Mix, AB Applied Biosystems, Warrington, UK) method was performed as previously described [19–21]. Specific primers (for selected genes such as IL-1*β*, Caspase11, Ccl20, Ccr1, Ccr5, Cd25 (IL-2r*α*), Cxcl10, Lpl, MMP12, Socs1, and Stat1) were designed according to the NCBI sequence database using the software Primer Expression 2.0 for testing selected genes. Hypoxanthine-guanine phosphoribosyltransferase (HPRT) was used as the housekeeping control. All PCR reactions were performed in triplicate. Relative mRNA levels for each sample were quantified using the 2^−∆∆*C*_t_^ approach and then normalized with respect to HPRT RNA as the standard.

## 3. Results

### 3.1. Gene Expression Profile of LNC (*Ex Vivo*) of HLXL-Treated, Vehicle-Treated, and Untreated (Control) Rats

Rats with AA were treated with either HLXL or vehicle (Water) for 7 d. Thereafter, their draining LNC were subjected to genomic analysis with the bead array system (Illumina). No significant difference (*P* > 0.05) was found between HLXL-treated (T_H_) and Water-treated group (T_W_), (data not shown). However, a comparison of T_H_/T_W_ group each with T_0_ group showed significant differences between T_H_ and T_W_ (Table 1). The expression of 152 genes was significantly altered when comparing T_H_ and T_0_ groups. Of these 152 genes, the expression of 7 genes was upregulated, while that of 145 genes was downregulated. In comparison, the expression of 246 genes was significantly modulated when comparing T_W_ and T_0_ groups. Of these, only 4 genes were upregulated. Furthermore, 126 genes were shared between T_W_ and T_H_. Various functional groups of DEG are summarized in Table 1. 

### 3.2. Gene Expression Profile of Antigen-Restimulated LNC (*In Vitro*)

#### 3.2.1. Bhsp65-Induced Gene Expression Profile of Water-Treated (T_W_) Arthritic Rats

**Figure 1:**
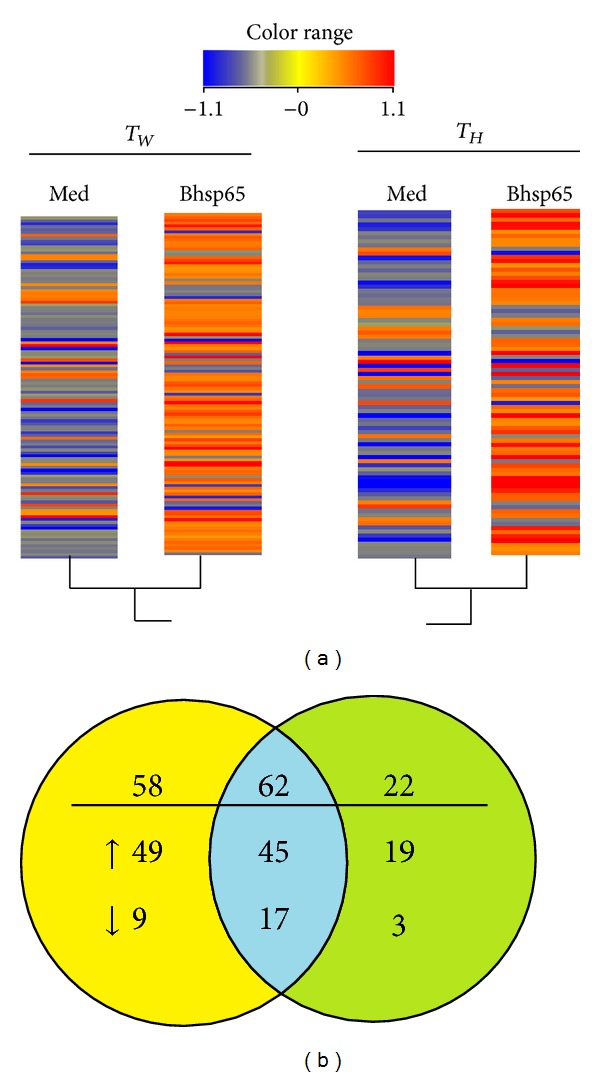
HLXL alters Bhsp65-induced gene expression in LNC. The draining lymph nodes were harvested from HLXL-treated (T_H_) and vehicle (Water-) treated (T_W_) arthritic LEW rats after 7 days of treatment. The lymph node cells (LNC) were cultured in vitro with or without Bhsp65. Thereafter, total RNA was extracted from cells and then used for further generation of cRNA for testing in microarrays. The gene expression profiles of Bhsp65-restimulated LNC are represented by heat map (a) and Venn diagram (b). LNC cultured in medium alone (*ex vivo*) served as the baseline for the Bhsp65-restimulated group. In heat map, red color indicates increased expression, whereas blue color indicates reduced expression of genes compared with the level of the corresponding baseline gene expression. DEG were selected using as cut-off fold change >2.0 and *P* value <0.05 corrected with Benjamin-Hochberg (Med: medium; Bhsp65: mycobacterial heat shock protein 65).

**Figure 2:**
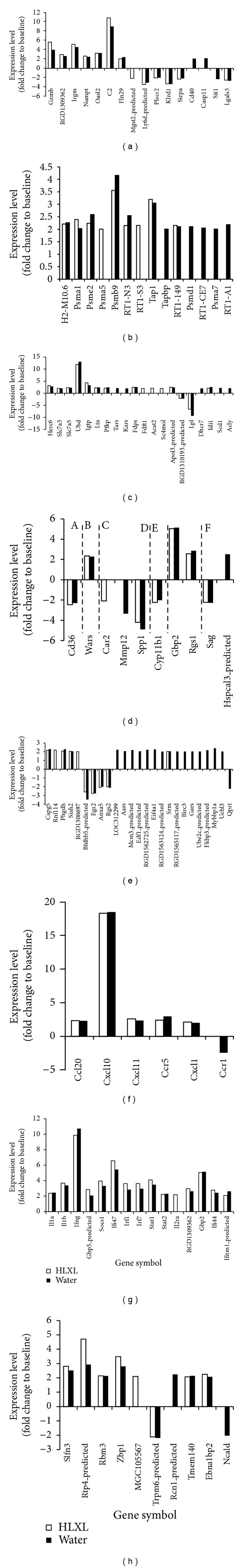
Comparison of the Bhsp65-induced DEG in lymphoid cells of HLXL-treated and Water-treated rats with AA. DEG were categorized into subgroups according to their biological function as follows: (a) general immune activity; (b) antigen processing and presentation; (c) metabolism; (d) other nonimmune related mechanisms involved in arthritis development: adhesion molecule (A), angiogenesis (B), bone damage (C), cellular response to oxygen and oxidation-reduction (D), signal transduction and pathways (E), and autoimmune disease-relevant antigen (F); (e) cell proliferation; (f) chemokines and receptors; (g) cytokines and receptors; and (h) proteins with binding activity (e.g., binding to actin, DNA, etc.). The level of gene expression is presented as fold change above 2, and *P* < 0.05.

A total of 120 genes were differentially regulated (≥2-fold and *P* < 0.05) upon antigen restimulation of LNC in Water-treated rats [21], of which 94 were upregulated and 26 were downregulated (Figure 1). The Gene Ontology classification of the DEG revealed 10 functional categories (Table 2). Based on their function, the three major categories of genes include immune response-related genes (48 genes, 40%), cell proliferation and apoptosis (17 genes, 14.2%), and genes with undefined function (25 genes, 21%). In the immune response-related genes, the 48 differentially expressed transcripts (Table 2) were linked to inflammation and chemotaxis, to the JAK-STAT cascade, and to antigen processing and presentation. The genes for three inflammatory cytokines, IL-1*β*, IL-1*α*, and IFN-*γ*, showed increased expression with fold change of 3.37, 2.43, and 10.73, respectively (Figure 2). The chemotaxis-related genes included several upregulated genes such as Cxcl10 (18.46-fold), Ccl20 (2.30-fold), Cxcl1 (2.0-fold), Cxcl11 (2.35-fold), and Ccr5 (2.98-fold) but one downregulated gene Ccr1 (−2.42-fold). Also identified were genes relating to protein lysis, and antigen processing and presentation were identified. In the cell growth and proliferation category, DEG were linked to DNA replication, RNA transcription, protein translation, regulation of apoptosis and cell proliferation, and posttranslational protein modification.

#### 3.2.2. Bhsp65-Induced Gene Expression Profile of HLXL-Treated (T_H_) Arthritic Rats

Microarray data analysis identified 84 genes that were differentially expressed in response to Bhsp65 in HLXL-treated group (Figure 1, Table 2). Of these 84, most of the transcripts (64 genes, 76.2%) were upregulated, while a relatively smaller group of genes (20 genes, 23.8%) was downregulated. As in the control group, the genes associated with immune response (40 genes, 47.6%) formed the major category in HLXL-treated group, followed by those with undefined function (13 genes, 15.5%) and metabolism (11 genes, 13.1%). Compared to Water (vehicle), HLXL affected specific genes in the same subgroup or the same gene (shared DEG) but to a different extent quantitatively. For example, HLXL treatment affected the category of immune response-related genes (inflammation and cytokines, chemotaxis, the JAK-STAT cascade, and antigen processing and presentation) as also observed following treatment with Water (Figure 2). But besides IL-1*β*, IL-1*α* and IFN-*γ* were significantly modulated by HLXL. IL-2r*α* was also upregulated in HLXL-treated group but not in control group.

#### 3.2.3. Comparison of Bhsp65-Induced Gene Expression in HLXL-Treated versus Water-treated Rats

 A comparison of the gene expression profiles of the two groups of rats revealed three sets of genes (Figure 1). Briefly, 22 genes were modulated mainly by HLXL, of which 19 were upregulated, but 3 were downregulated. Another 58 genes were altered exclusively in control rats. Of these, 49 showed increased expression, while 9 showed reduced expression. A set of 62 genes were shared between the two groups. Of these, 45 were upregulated, while 17 were downregulated. We then examined the relative expression of specific genes grouped accordingly to the role of their encoded proteins in particular immunobiological processes (Figure 2). This analysis revealed multiple target genes whose expression was differentially up-/downregulated in HLXL-treated versus Water-treated rats. These genes represent multiple processes involved in the pathogenesis of arthritis, namely, general immune activity, cellular proliferation, antigen processing and presentation, adhesion molecules, chemokines and their receptors, cytokines and their receptors, angiogenesis, bone remodeling, and signal transduction and pathways.

### 3.3. Validation of Expression of Genes by qPCR

**Figure 3:**
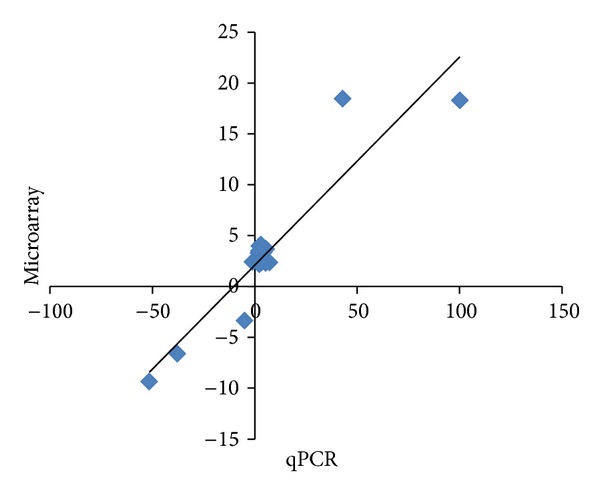
Verification of microarray data by qPCR. The expression of IL-1*β*, Caspase11, Ccl20, Ccr1, Ccr5, Cd25 (IL-2r*α*), Cxcl10, Lpl, MMP12, Socs1, and Stat1 identified through microarray analysis was further verified by qPCR. An analysis of the correlation between the levels of gene expression determined by microarray and qPCR was performed. The coefficient of correlation (*r* ^2^) was 0.8671, which was statistically significant (*P* < 0.05).

The qPCR results supported the microarray data (Figure 3), with differences only in the scale of estimated upregulation or downregulation. A significant correlation was observed in the expression of 11 genes (described under Section 2.7) (*r* ^2^ = 0.8610, *P* < 0.05).

## 4. Discussion

In our previous studies on testing HLXL in rats with AA, we showed that HLXL suppressed the ongoing disease via downregulation of proinflammatory cytokines (IL-1*β*, IL-6, IL-17, and IFN-*γ*), chemokines (RANTES, MCP-1, MIP-1*α*, and GRO/KC), and biomolecules involved in cartilage and bone damage such as matrix metalloproteinase (MMP) [4–6]. However, these parameters represent only a small proportion of mediators of various effector pathways involved in the pathogenesis of arthritis. Furthermore, this limited set of parameters cannot fully explain the mechanism of action of HLXL that contains 11 herbs. Microarray-based gene expression profiling has enabled us to explore the complex biological processes regulated at the transcriptional level, and therefore, it is an appropriate tool to study the antiarthritic activity of HLXL.

Analysis of the gene expression profiles of LNC restimulated with Bhsp65 in vitro revealed several interesting sets of DEG when comparing HLXL-treated versus Water-treated groups. In contrast, no significant difference was observed in gene expression profile of LNC tested ex vivo (without Bhsp65 restimulation). The results of in vitro analysis shed light on alteration of gene expression in LNC by HLXL. Downregulation of genes associated with antigen processing and presentation was the most prominent effect of HLXL treatment when compared to its vehicle (Water). Furthermore, HLXL-mediated downregulation of the expression of the costimulatory molecule CD40 suggests reduced activation of T cells. This represents one of the ways to correct the overactive immune activity in AA. After activation, T cells proliferate leading to expansion of the T-cell pool, followed by apoptosis of most of the activated T cells. T-cell proliferation is an important process to mediate effective immunity, but excessive proliferation accompanies autoimmunity [22]. Our present work illustrates the ability of HLXL to downregulate immune cell activation and proliferation in AA. Reduced expression of genes linked to DNA replication (Mcm3_predicted), RNA transcription (Mybbp1a, Bhlhb5_predicted, and Edf1), amino acid transportation (Tars, Kars), translation or synthesis of protein (Eif4a1, RGD1565900_predicted, RGD1561310_predicted, and RGD1565117_predicted), posttranslational protein modification and folding (Ube2c_predicted, Fkbp3_predicted), pro-/antiapoptosis (RGD1308697, Birc3), and cell differentiation (Rnf114) affected by HLXL indicated the inhibitory role of HLXL in antigen-induced cellular activation and proliferation.

Taken together, HLXL mainly targeted three pathways to exert its function in inhibiting AA: first, by suppressing the activation of immune cells; second, by inhibiting the proliferation of immune cells; and third, by controlling the pro-inflammatory cytokine IFN-*γ*. Th1 cells and the characteristic cytokine (IFN-*γ*) secreted by them play a critical role in the pathogenesis of RA [7]. In this context, downregulation of IFN-*γ* by HLXL is of significance.

No significant difference in gene expression profiles of LNC tested ex vivo without antigenic restimulation was observed when HLXL-treated group was compared with Water-treated group. We suggest that this finding relates to the dynamics of gene expression during the course of AA. In the AA model, most significant changes in gene expression occur during the incubation phase of AA, for example, on d 7 after disease induction [19]. Thereafter, there is marked reduction in gene expression, returning to the normal level during the peak (d 18) and recovery (d 25) phases of AA. In this study, the rats developed arthritis on d 12 and then were treated with HLXL or vehicle for 7 d. The time point of d 19 equals the peak phase of AA, and by then, most of the gene expression changes return to normal (baseline) levels. This might explain why the effect by HLXL was not detectable when LNC were tested ex vivo without Bhsp65 restimulation.

Our results showed a protective effect of HLXL against AA and demonstrated that HLXL treatment results in changes in Bhsp65-induced gene expression in LNC compared to Water treatment. Taken together, the major findings in regard to HLXL-induced changes can be summarized as follows: (a) The maximum changes were observed in immune activity-related genes, including an overexpression of innate/adaptive immune response genes and downregulation of genes involved in inflammatory response, antigen processing and presentation, costimulation, and IFN-*γ* response genes. (b) The expression of cell proliferation genes was reduced coupled with increased expression of genes related to apoptosis, which might be a protective response targeted against pathogenic effector cells. (c) Alleviation of oxidation stress response might be one of the protective mechanisms of HLXL as supported indirectly by our results. (d) HLXL treatment upregulated the expression of genes encoding interleukin-2 receptor alpha (IL-2Ra; CD25) and suppressor of cytokine signaling 1 (SOCS1). CD25 is component of the multimeric IL-2 receptor that binds the cytokine IL-2, whereas SOCS1 is a regulator of both Janus kinase/signal transducer and activator of transcription (JAK/STAT) and IFN-*γ*-STAT1 signaling pathways. Interestingly, both of these molecules play an important role in the development, expansion, and activity of CD4^+^CD25^+^Foxp3^+^ regulatory T cells (Treg) [23–26]. Treg belong to two categories, natural (thymic derived) and induced (peripherally-derived) [23–26]. Treg are effective suppressors of pathogenic effector T cells of T helper 17 (Th17) and Th1 lineage, both of which have been invoked in the pathogenesis of autoimmune arthritis in different experimental models as well as patients with RA. Natural Treg constitutively express CD25 and Forkhead box p3 (Foxp3), whereas induced Treg express it along with induction of Foxp3 expression when they develop from CD4^+^CD25^−^ T cells under appropriate conditions of T-cell activation [23–26]. The above-mentioned HLXL-induced changes also represent the likely mechanisms by which HLXL exerts its antiarthritic effect in rats with AA. Further characterization of these molecular pathways would be helpful in advancing our understanding of the pathogenesis of arthritis and in identifying new prognostic biomarkers as well as novel therapeutic targets for RA.

## Figures and Tables

**Table 1:** The gene expression profiles of LNC (*ex vivo*) of HLXL-/Water-treated rats compared with Untreated rats.

Functional group	Water-treated (T_w_)		HLXL-treated (T_H_)	
	Individual-DEG	Shared-DEG		Individual-DEG
Cell proliferation and apoptosis	24	33		3
Immune activity	16	13		3
Metabolism				
Lipid metabolism	0	1		1
Oxidation-reduction	0	6		5
Other metabolic processes	5	4		0
Binding activity	14	9		2
Transporter	2	4		2
Signal transduction	2	4		0
Other function	7	5		0
Undefined function	50	47		10

Sub total	120	126		26
Total	246		152	

The gene expression profile of Water-treated (T_w_) or HLXL-treated (T_H_) group versus that of untreated arthritic rats (T_0_). Functional category is based on biological processes. DEGs were selected using as cut off fold change >2.0 and *P* < 0.05 corrected with Benjamin-Hochberg.

**Table 2:** The gene expression profiles of Bhsp65-restimulated LNC of HLXL-treated and vehicle (Water)-treated rats.

Gene function	HLXL-treated		Water-treated	
	UR-DEG	DR-DEG	UR-DEG	DR-DEG
Immune response-related	36	4	41	7
Cell proliferation and apoptosis	3	3	13	4
Binding activity	6	0	5	1
Metabolism				
Lipid metabolism-related processes	6	2	6	2
Other metabolic processes	2	1	3	1
Undefined	7	6	19	6
Angiogenesis	1	1	1	1
Signal transduction and pathways	1	2	1	2
Autoantigen	0	0	1	0
Others	0	0	3	0
Transport	2	1	1	2

Sub-total	64	20	94	26
Total	84		120	

UR: upregulated; DR: downregulated; DEG: differentially expressed genes. DEG in response to Bhsp65-restimulation of LNC were compared. The gene expression profile of LNC cultured with Bhsp65 was compared with that of LNC in medium (med) using the cut off *P* < 0.05 corrected with Benjamin-Hochberg, fold change >2.0 as described in Section 2.6. The functional categories of these DEG were based on the main biological processes involving them.
